# LAT Region Factors Mediating Differential Neuronal Tropism of HSV-1 and HSV-2 Do Not Act in *Trans*

**DOI:** 10.1371/journal.pone.0053281

**Published:** 2012-12-31

**Authors:** Andrea S. Bertke, Kathleen Apakupakul, AyeAye Ma, Yumi Imai, Anne M. Gussow, Kening Wang, Jeffrey I. Cohen, David C. Bloom, Todd P. Margolis

**Affiliations:** 1 Francis I. Proctor Foundation and Department of Ophthalmology, University of California San Francisco, San Francisco, California, United States of America; 2 Department of Molecular Genetics and Microbiology, University of Florida College of Medicine, Gainesville, Florida, United States of America; 3 Medical Virology Section, Laboratory of Infectious Diseases, National Institute of Allergy and Infectious Diseases, Bethesda, Maryland, United States of America; McMaster University, Canada

## Abstract

After HSV infection, some trigeminal ganglion neurons support productive cycle gene expression, while in other neurons the virus establishes a latent infection. We previously demonstrated that HSV-1 and HSV-2 preferentially establish latent infection in A5+ and KH10+ sensory neurons, respectively, and that exchanging the latency-associated transcript (LAT) between HSV-1 and HSV-2 also exchanges the neuronal preference. Since many viral genes besides the LAT are functionally interchangeable between HSV-1 and HSV-2, we co-infected HSV-1 and HSV-2, both *in vivo* and *in vitro*, to determine if *trans*-acting viral factors regulate whether HSV infection follows a productive or latent pattern of gene expression in sensory neurons. The pattern of HSV-1 and HSV-2 latent infection in trigeminal neurons was no different following co-infection than with either virus alone, consistent with the hypothesis that a *trans*-acting viral factor is not responsible for the different patterns of latent infection of HSV-1 and HSV-2 in A5+ and KH10+ neurons. Since exchanging the LAT regions between the viruses also exchanges neuronal preferences, we infected transgenic mice that constitutively express 2.8 kb of the LAT region with the heterologous viral serotype. Endogenous expression of LAT did not alter the pattern of latent infection after inoculation with the heterologous serotype virus, demonstrating that the LAT region does not act in *trans* to direct preferential establishment of latency of HSV-1 and HSV-2. Using HSV1-RFP and HSV2-GFP in adult trigeminal ganglion neurons *in vitro,* we determined that HSV-1 and HSV-2 do not exert *trans*-acting effects during acute infection to regulate neuron specificity. Although some neurons were productively infected with both HSV-1 and HSV-2, no A5+ or KH10+ neurons were productively infected with both viruses. Thus, *trans*-acting viral factors do not regulate preferential permissiveness of A5+ and KH10+ neurons for productive HSV infection and preferential establishment of latent infection.

## Introduction

Following peripheral inoculation with either herpes simplex virus 1 (HSV-1) or HSV-2, the virus gains access to the axons of sensory neurons and is transported in a retrograde fashion to neuronal cell bodies in primary sensory ganglia, where infection follows either a productive or latent pathway of viral gene expression. In some neurons, productive cycle viral genes are expressed and progeny virus is produced, while in other neurons, the productive cycle fails to progress and the virus establishes a latent infection [1]–[3]. The factors that determine whether HSV progresses through the productive cycle or establishes a latent infection in specific neurons are not clear, although there is evidence that the latency-associated transcript plays a role in this process [2], [3].

In previous *in vivo* studies, we demonstrated that HSV-1 and HSV-2 preferentially establish latency and express LAT in specific populations of neurons within the trigeminal ganglia (TG) [1], [2]. Primary sensory neurons are a diverse population of cells that can be classified according to cellular morphology, physiological response properties, and patterns of gene expression. Some neuronal populations of the trigeminal ganglion (TG) are much less permissive for productive viral infection than others, and permissiveness differs between HSV-1 and HSV-2. The neuronal population identified by mAb A5 is relatively non-permissive for HSV-1 productive infection *in vitro*, compared to other types of sensory neurons, and also serves as the principal reservoir of latent HSV-1 *in vivo* [1], [4]. In contrast, A5+ neurons are relatively permissive for HSV-2 productive infection *in vitro* [*4*]. The neuronal population identified by mAb KH10 is relatively non-permissive for HSV-2 productive infection *in vitro* and serves as the principal reservoir of latent HSV-2 *in vivo* [2], [4]. Thus, productive infections with HSV-1 and HSV-2 are regulated differently in A5+ and KH10+ neurons. Latent infection with HSV may simply be the default pattern of viral gene expression in a neuron that is non-permissive for productive infection.

Studies have shown that a number of different viral functions map to the HSV-1 LAT region, including virulence, establishment of latency, reactivation from latency, and inhibition of host cell apoptosis (reviewed in [5] & [6]). Studies using chimeric viruses have suggested that preferential establishment of latency in specific types of trigeminal ganglion neurons maps to this same region of the viral genome [2], [3]. However, the functional mapping of the HSV-2 region is less clear, as the LAT sequence differs considerably from that of HSV-1. The primary LAT transcript is transcribed from the repeat regions of both viral genomes, antisense to the genes coding for the immediate-early (IE) transactivator ICP0 and the PKR inhibitor ICP34.5. The IE transactivator, ICP4, is also transcribed from the repeat regions, just downstream of the 3′ end of the LAT primary transcript. The unstable primary LAT transcript is spliced into a stable 2 kb intron, although the intron itself does not appear to be directly involved in the latency-related functions attributed to LAT [6]–[8]. MicroRNAs are produced from the LAT region of both HSV-1 and HSV-2 viral genomes and it has been suggested that these microRNAs may play a role in regulating expression of ICP0, ICP34.5, and ICP4, key regulatory proteins in HSV productive cycle gene expression [9]–[11]. Although various mechanisms have been proposed for the function of the LAT region, it is still unclear whether the LAT functions in a *cis-*acting or *trans-*acting manner. Understanding the different mechanisms that regulate productive vs. latent infection in different populations of TG neurons is essential to effectively inhibit productive infection of latent viral reservoirs. In the current study, we tested whether *trans*-activating viral factors, especially from the 5′ end of the LAT region of the viral genome, regulate whether HSV infection will follow either a productive or latent pattern of viral gene expression in specific types of TG neurons.

## Materials and Methods

### Viruses

The wild type HSV-1 strains KOS and 17syn+ and the wild type HSV-2 strains 333 and MS were propagated in rabbit skin cells [1]. HSV1-VP26-RFP and HSV2-VP26-GFP were constructed by homologous recombination of plasmid pK26GFP [12] with wild type HSV-1 (17syn+) and HSV-2 (333) viral DNA, as described previously [4]. Viral titers were determined by standard viral plaque assay on Vero cells.

### Animals and Inoculations

For dual infection with HSV-1 and HSV-2, six week-old female Swiss Webster mice (Simonsen Laboratories, Gilroy, CA) were anesthetized by intraperitoneal injection with sodium pentobarbital followed by topical administration of 0.5% proparacaine hydrochloride. Following corneal scarification, eyes were infected with a 10 µl inoculum containing both HSV-1 and HSV-2 at titers designated in Tables 1 and 2. Forty hours after inoculation, 1.2 mg/ml acyclovir (ACV) was added to the drinking water, a time point that permits both productive viral infection of the trigeminal ganglia (TG) and the establishment of viral latency but promotes the survival of the mice. TG were harvested at 21 days post-inoculation and frozen sections were collected as previously described [3]. For the study of HSV infection of LAT transgenic mice, corneas of HSV-1 LAT transgenic mice *LAT 3549* [13], HSV-2 LAT transgenic mice LATpa *5238* [14] or C57BL/6 were scarified and inoculated with HSV-1 (17syn+) or HSV-2 (333). Acyclovir was added to the drinking water only for those mice infected with HSV-2 strain 333. TG were harvested at day 21–28 and frozen sections were collected as previously described [3].

**Table 1:** Distribution of latently infected neurons in mouse trigeminal ganglia following ocular inoculation with mixed HSV-1 and HSV-2.

Virus	FISH LAT probe	A5+ neurons	KH10+ neurons
KOS 10^6^ and 333 10^6^	HSV-1	27.6% (58/210)	2.3% (5/215)
	HSV-2	4.3% (20/470)	55.7% (241/433)
KOS 10^8^ and 333 10^6^	HSV-1	29.6% (94/328)	5.6% (20/354)
	HSV-2	2.2% (7/319)	48.6% (104/214)
KOS 10^7^ and 333 10^5^	HSV-1	43.0% (102/237)	2.0% (4/202)
	HSV-2	7.2% (22/306)	41.5% (85/205)
KOS 10^6^	HSV-1	29.7% (120/404)	3.0% (8/266)
KOS 10^7^	HSV-1	27.2% (64/235)	2.2% (5/224)
KOS 10^8^	HSV1	31.6% (65/206)	12% (47/379)**
333 10^5^	HSV-2	7.6% (21/277)	43.1% (109/253)
333 10^6^	HSV-2	3.2% (15/474)	52.0% (156/300)
17syn+10^8^ and MS 10^8^	HSV-1	40.2% (99/246)	3.2% (15/476)
	HSV-2	9.5% (37/391)	46.5% (166/357)
17syn+10^8^	HSV-1	48.7% (75/154)	4% (10/164)**
MS 10^8^	HSV-2	5.0% (11/219)	52.1% (182/349)*

Twenty-one days after ocular inoculation, latently infected trigeminal ganglia were sectioned and assayed by combined FISH/IF for HSV-1 or HSV-2 LAT and neuronal cell markers. The percentage of LAT+ neurons that co-labeled with monoclonal antibodies A5 or KH10 is presented. The raw data are also presented (dual-labeled neurons/number of LAT-positive neurons evaluated).

* *Previously published data presented for the purposes of comparison only* [*3*] *.*

** *Previously published data presented for the purposes of comparison only* [*2*] *.*

**Table 2:** Distribution of latently infected neurons after cross-serotype infection of LAT-expressing transgenic mice.

Virus	Mouse Strain	A5+ neurons	KH10+ neurons
HSV-1 17syn+	LATpa 5238 (HSV-2 LAT transgenic)	48.0% (110/229)	4.1% (9/217)
	C57BL/6 (wildtype)	51.5% (87/169)	3.1% (9/293)
HSV-2 333	LAT 3549 (HSV-1 LAT transgenic)	3.1% (16/516)	45.5% (106/233)
	C57BL/6 (wildtype)	2.1% (5/235)	42.3% (63/149)

Twenty-one or twenty-eight days after ocular inoculation sections of latently infected trigeminal ganglia were assayed by combined FISH/IF. The percentage of LAT-positive (specific for infected HSV type) neurons that co-labeled with monoclonal antibodies A5 or KH10 is presented. The raw data are also presented (dual-labeled neurons/number of LAT-positive neurons evaluated).

### Ethics Statement

All animal studies were approved and conducted according to the policies and guidelines of the respective Institutional Animal Care and Use Committees of the University of California San Francisco, National Institute of Allergy and Infectious Diseases, and the University of Florida College of Medicine.

### *In vitro* Neuronal Cultures and Infections

Trigeminal ganglia were removed from 6 week old Swiss Webster mice, dissociated enzymatically with papain, collagenase, and dispase, enriched for neurons through an Optiprep gradient, and plated on poly-D-lysine/laminin coated 8-well chamber slides (BD Biosciences) at a density of 3000 neurons/well as previously described [4]. Cultures were maintained in complete neuronal medium consisting of Neurobasal A medium supplemented with 2% B27, 1% penicillin-streptomycin, L-glutamine, nerve growth factor (NGF), glial cell line-derived neurotrophic factor (GDNF), and neurturin (NTN), as described previously [4]. Fluorodeoxyuridine and aphidicolin were added for the first 3 days to inhibit residual non-neuronal cell proliferation. Cultures were infected at multiplicities of infection (MOI) of 30 or 10 in Neurobasal A medium. After a one-hour adsorption period, virus was removed and replaced with complete neuronal medium (without mitotic inhibitors). At 10 hours post-inoculation, cells were fixed by adding paraformaldehyde (PFA) directly to the media at a final concentration of 2% for 5 minutes, followed by immunofluorescent staining with A5 and KH10 monoclonal antibodies (mAbs).

### Combined Staining by Fluorescent *in situ* Hybridization (FISH) and Immunofluorescence (IF)

HSV-1 LAT-specific probe and HSV-2 LAT-specific probe were prepared by using DIG RNA Labeling Mix (Roche), and combined staining for LAT RNA and neuronal cell markers was carried out as previously described [2], [3], [15]. HSV-1 and HSV-2 LAT probes were tested on sections of ganglia infected with either KOS or 333 and no cross-reactivity was observed.

## Results

### Preferential Establishment of HSV-1 and HSV-2 Latent Infections are not Regulated by *trans-*acting Factors

We previously demonstrated that HSV-1 preferentially establishes latent infection of murine sensory ganglia in A5+ neurons and HSV-2 preferentially establishes latent infection in KH10+ neurons, as identified by dual fluorescent *in situ* hybridization (FISH) for LAT and immunofluorescent (IF) staining with mAbs A5 and KH10 for neuronal markers [2], [3]. Given that several viral factors are functionally interchangeable between HSV-1 and HSV-2, we hypothesized that any *trans-*acting viral factors involved in regulating neuronal preferences that are produced by one viral species, may be capable of directing a phenotypic change in the preferential establishment of latency of the other viral species, permitting identification of *trans-*acting factors involved in neuron specificity. If we were able to identify such a *trans-*acting factor, we could potentially identify neuron-specific pathways involved in regulating productive HSV infection in neurons. Therefore, we co-infected mouse corneas with equivalent titers of both HSV-1 and HSV-2, either 10^6^ or 10^8^ pfu/ml in 10 µl media. Twenty-one days after ocular inoculation, tissue sections of latently infected trigeminal ganglia (TG) were assayed by combined FISH for LAT and IF for the A5 and KH10 neuronal markers.

In the TG of mice that had been co-infected with a mixture of KOS and 333 at 10^6^ pfu/ml, 27.6% of the HSV-1 LAT+ neurons co-labeled with mAb A5 and 2.3% of HSV-1 LAT+ neurons co-labeled with mAb KH10+ (Table 1). In these same ganglia, only 4.3% of the HSV-2 LAT+ neurons co-labeled with mAb A5, but 55.7% of HSV-2 LAT+ neurons co-labeled with mAb KH10+. These results were very similar to what was observed in the ganglia of control mice infected with either KOS or 333 alone at 10^6^ pfu/ml (Table 1). These results were also very similar to what we reported previously following ocular inoculation with either KOS or 333 at higher viral titers [1]–[3]. It should be noted that the titer of the viral inocula used in these studies was lower than that used in our previously reported studies. Co-infection with higher viral titers resulted in death of the experimental animals, even though infected mice were treated with acyclovir (ACV) starting at 40 hours post inoculation. We conclude that the pattern of HSV-1 (KOS) and HSV-2 (333) latent infection in neurons of the mouse trigeminal ganglion is no different following simultaneous co-infection than it is following infection with either virus alone; HSV1 preferentially establishes latent infection in A5+ neurons and HSV-2 preferentially establishes latent infection in KH10+ neurons. Therefore, we were unable to identify functional *trans-*acting factors that affect preferential establishment of latent infections with HSV-1 KOS and HSV-2 333.

We next considered the possibility that a *trans*-acting effect on the HSV latency phenotype might only be observed if we infected with an excess of one virus. To accomplish this, we co-infected mice with 100-fold greater quantities of one HSV strain than of the other. Mice infected with 100-fold greater quantities of HSV-2 compared to HSV-1 did not survive at viral inocula that permitted HSV-1 to establish latent sites of infection detectable by FISH for LAT. However, mice infected with excess HSV-1 did survive, and as summarized in Table 1, co-infection with 2-log excess HSV-1 (at either 10^8^ or 10^7^ pfu/ml) had no effect on the latency phenotype of HSV-2. Thus, we were unable to identify functional *trans-*acting factors that affect preferential establishment of latent infections with HSV-1 KOS and HSV-2 333. These data also suggest that neuronal preferences are not the result of a competition or dominance effect of one virus over the other, since we likely would have observed some change in phenotype in A5+ and/or KH10+ neurons if our results were simply a dominant-recessive interaction.

Different strains of HSV-1 and HSV-2 can have very different phenotypes. To investigate whether strains of HSV-1 and HSV-2 other than KOS and 333 express functional *trans*-acting factors capable of crossing viral serotypes, we co-infected mice with HSV-1 (17syn+) and HSV-2 (MS). As summarized in Table 1, the results were similar to what we observed following co-infection with KOS and 333. Co-infection appeared to have little or no effect on the latency phenotype of HSV-1 (17syn+) or HSV-2 (MS). HSV-1 17syn+ preferentially established latency in A5+ neurons and HSV-2 MS preferentially established latency in KH10+ neurons, almost identically to results from mice infected with either 17syn+ or MS alone (Table 1). In this study we were able to infect at higher titers of virus without death of experimental animals, presumably due to the relatively lower virulence of MS as compared to 333. Although we also carried out co-infections with 17syn+ and 333, these mice only survived when the viral titers were so low that only rare LAT+ neurons could be found in the TG of infected mice.

In summary, the results that we obtained following simultaneous co-infection of mice with HSV-1 and HSV-2 are consistent with the hypothesis that a *trans*-acting viral factor is not responsible for the different patterns of latent infection of HSV-1 and HSV-2 in A5+ and KH10+ neurons. One could argue that our results were simply due to failure to co-infect individual nerve endings on the ocular surface with both HSV-1 and HSV-2. However, this is unlikely since the infections were carried out with a very high concentration of mixed viral inoculum and there is no preferential uptake and axonal transport of HSV into A5+ and KH10+ neurons (9). Nevertheless, to more definitively rule out alternative explanations for our findings, we carried out additional studies using LAT transgenic mice and dissociated TG neuron cultures.

### Endogenous Expression of HSV-1 LAT in *trans* does not Affect the Neuronal Subtype Preference of HSV-2 Latency and Vice Versa

We previously demonstrated that a 2.8 kb region of the LAT gene directed differential latent infection in A5+ and KH10+ neurons, by swapping this region of the LAT between HSV-1 and HSV-2 in chimeric viruses [2], [3]. Previous studies have shown that this same LAT region expressed in transgenic mice did not influence the establishment of latency when the mice were infected with HSV of the homologous serotype (i.e. HSV-1 LAT-expressing mice infected with HSV-1 [13] or HSV-2 LAT-expressing mice infected with HSV-2 [14]). Since this 2.8 kb LAT region in the context of the chimeric viruses *did* influence differential latent infection, we hypothesized that a *trans*-acting factor expressed by the LAT transgene in the mice should be able to direct differential latent infection if the mice were infected with the heterologous virus serotype (i.e. HSV-1 LAT-expressing mice infected with HSV-2). To determine if this region of LAT exerts an effect on the virus-specific preferential establishment of latency in *trans*, we studied the pattern of latent HSV infection in the trigeminal ganglia of transgenic mice that constitutively express this 2.8 kb region of LAT in neurons *in vivo*. In these transgenic mice, LAT is expressed in at least 85–90% of the trigeminal neurons, and the LAT levels in these neurons are at least as high as that observed during latent HSV infection (8,20); thus heterologous LAT is expressed in at least 85–90% of neurons that become infected after corneal uptake and axonal transport. Furthermore, this system takes advantage of the fact that LAT is expressed prior to infection, thus maximizing the chance of seeing a *trans* effect of LAT on the phenotype of HSV latency, regardless of any temporal association that may occur.

Mice expressing the HSV-1 LAT transgene (LAT 3549) [13] were infected by ocular inoculation with HSV-2 (333) and mice expressing the HSV-2 LAT transgene (LATpa 5238) [14] were infected by ocular inoculation with HSV-1 (17syn+). Twenty-one or twenty-eight days after inoculation, latently infected TGs were analyzed for HSV LAT expression and A5 and KH10 neuronal markers by combined FISH/IF (Table 2). We have previously demonstrated that our FISH probes for LAT are type-specific [2]. In the HSV-2 LAT transgenic mice infected with HSV-1, 48.0% of the HSV-1 LAT+ neurons were A5+, while only 4.1% were KH10+, similar to the results after HSV-1 infection of the parental mouse strain (C57BL/6). In the HSV-1 LAT transgenic mice infected with HSV-2, only 3.1% of the HSV-2 LAT+ neurons were A5+, whereas 45.5% were KH10+, similar to the results after HSV-2 infection of the parental mouse strain (C57BL/6). These findings are consistent with the hypothesis that this 2.8 kb region of the LAT does not act cross-serotype in *trans* to direct the preferential establishment of latency of HSV-1 and HSV-2 in different types of neurons. Furthermore, given the very high prevalence of LAT expression in the TG neurons of the LAT transgenic mice, it is very unlikely that a majority of the neurons infected by HSV were simply those in which the heterologous LAT was not highly expressed. We conclude that factors within this LAT region that regulate preferential establishment of latent infection are likely to be *cis*-acting factors.

### HSV-1 and HSV-2 do not Exert *trans*-acting Effects in Primary Neuronal Culture

**Figure 1:**
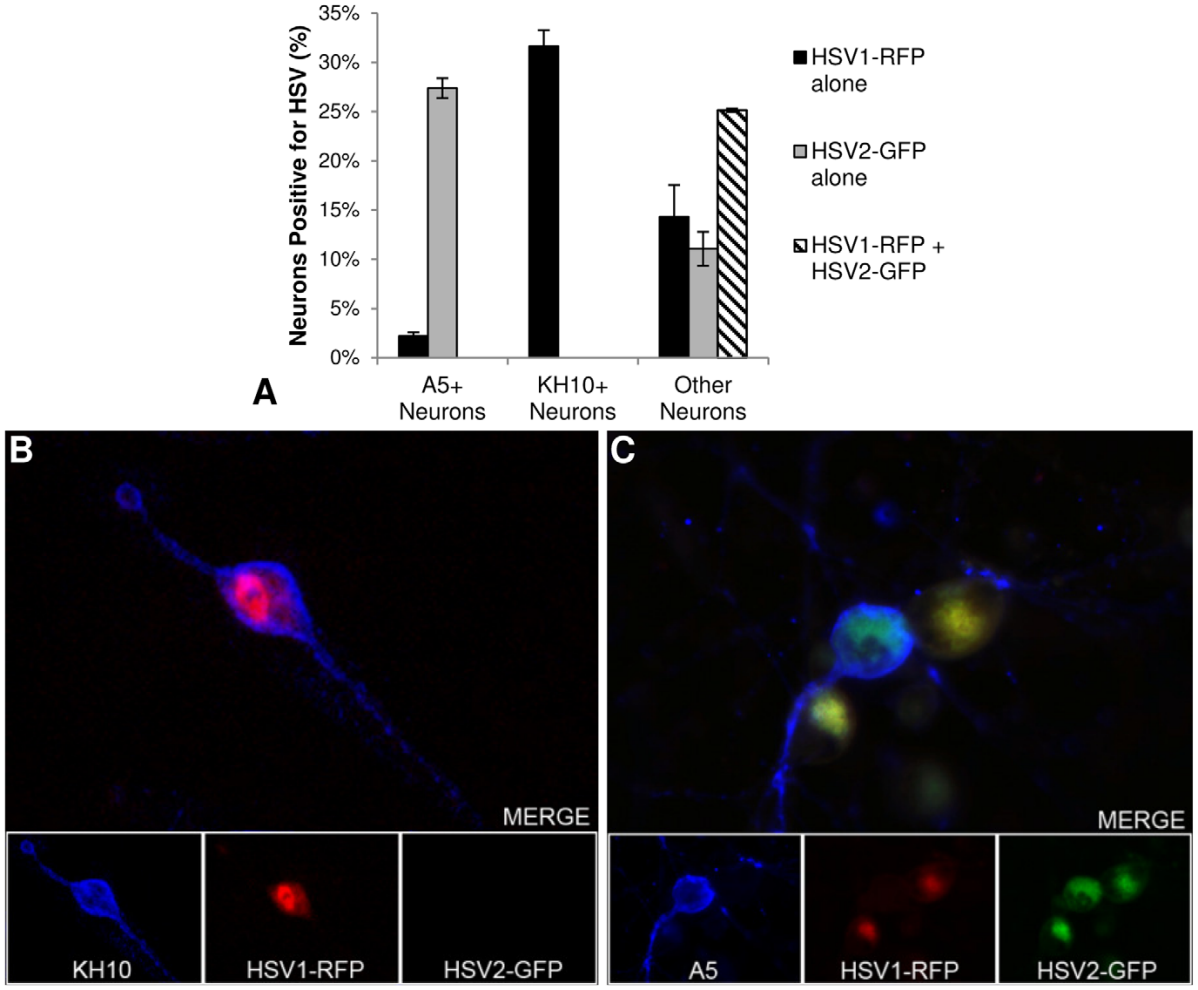
Co-infection of dissociated adult trigeminal neurons *in vitro.* Dissociated TG neurons were co-infected with HSV1-VP26-RFP and HSV2-VP26-GFP and the percentage of different neuronal populations (*A5+, KH10+, or “Other Neurons” not labeling with either mAb A5 or KH10) that were productively infected with HSV-1 and HSV-2 were assayed by fluorescence microscopy. A) Percentage of A5+, KH10+, and “Other” TG neuronal populations productively infected with HSV1 and HSV2. B) Representative image of KH10+ neuron positive for HSV1-RFP. C) Representative image of A5+ neuron positive for HSV2-VP26-GFP, flanked by two A5- neurons positive for both HSV1-RFP and HSV2-GFP.*

Evaluating acutely infected trigeminal ganglia for productive infection and neuronal markers simultaneously can be technically difficult due to the asynchronicity of acute neuronal infection in the ganglia, as well as the immune infiltrate and tissue destruction. We previously showed that cultured adult A5+ and KH10+ trigeminal ganglion neurons are non-permissive for productive infection of HSV-1 and HSV-2, respectively, which correlates with preferential establishment of latent infection in these specific types of neurons *in vivo*. We further demonstrated that this was not a consequence of a low rate of infection of the cultured neurons, since ∼90% of cultured neurons become infected, but that less than 30% of all infected neurons support a productive infection (1). To determine if HSV-1 and HSV-2 exert *trans*-acting effects during acute infection we infected dissociated adult murine trigeminal ganglia neurons *in vitro* with HSV1-RFP (17syn+ based virus), expressing red fluorescent protein as a VP26-RFP fusion protein, and HSV2-GFP (333-based virus), expressing a VP26-GFP fusion protein. Cultures were infected for 8, 10, or 15 hours at MOIs of 100, 30, and 10, after which they were immunostained with mAbs A5 or KH10 and evaluated for sites of productive infection, as detected by expression of GFP or RFP (data for 30 moi at 10 hpi are shown in Figure 1). Uninfected dissociated adult trigeminal ganglia neuronal cultures maintain heterogeneity of neuronal cell types *in vitro* and express the A5 and KH10 markers at proportions similar to those found *in vivo,* and expression of the VP26 fusion proteins by these viruses effectively represents productive infection [4]. When the cultures were infected with HSV-1 alone and analyzed 10 hours post inoculation (hpi) (the peak of productive infection), 31.7% of KH10+ neurons were productively infected, as detected by RFP expression, with only 2.2% A5+ neurons expressing RFP (Figure 1A). In contrast, when cultures were infected with HSV-2 alone and analyzed at the peak of productive infection at 10 hpi, 27.4% of A5+ neurons were productively infected as detected by GFP expression, with no KH10+ neurons expressing GFP. When the cultures were co-infected with both HSV-1 and HSV-2, the pattern of infection of A5+ and KH10+ neurons was almost identical to that observed in the control cultures that had been infected with either HSV-1 or HSV-2 alone. HSV-1 productively infected KH10+ neurons, as shown in Figure 1B, but rarely A5+ neurons, and HSV-2 productively infected A5+ neurons, as shown in figure 1C, but rarely KH10+ neurons. Furthermore, although 25.2% of all co-infected neurons expressed both GFP and RFP, indicative of productive infection of these neurons with both HSV-1 and HSV-2, no A5+ or KH10+ neurons were productively infected with both viruses (Figure 1A). Figure 1C shows two neurons in which HSV1-RFP and HSV2-GFP co-localized, adjacent to an A5+ neuron in which only HSV2-GFP is evident. These data demonstrate that although HSV-1 and HSV-2 are capable of productively co-infecting sensory neurons, the A5+ and KH10+ neurons are preferentially non-permissive for HSV-1 and HSV-2, respectively. Furthermore, the data strongly suggest that independent *trans*-acting viral factors do not regulate the preferential non-permissiveness of A5+ and KH10+ neurons for productive infection. If *trans*-acting factors were responsible and interchangeable between HSV-1 and HSV-2, we would have found dual productive infection of HSV-1 and HSV-2 in A5+ and KH10+ neurons.

## Discussion

Sensory ganglia, including the trigeminal ganglion, are collections of thousands of cells including a heterogeneous group of primary sensory neurons. When sensory ganglia are infected with HSV-1 or HSV-2, infected neurons follow either of two pathways of viral gene expression. In some neurons, a productive cycle of viral gene expression occurs while in other neurons, the virus establishes a latent infection [1]–[3], [15]. Not all of the neurons in sensory ganglia are equally susceptible to productive infection and specific types of neurons are more likely than others to harbor latent HSV-1 and HSV-2 [1], [2]. In previous studies, we have shown that HSV-1 and HSV-2 preferentially establish latent infection and express LAT in different subtypes of sensory neurons after ocular infection of mice [1]–[3]. Approximately 50% of the latent HSV-1 LAT+ sites are in A5+ neurons, while about 50% of the latent HSV-2 LAT+ sites are in KH10+ neurons. This preferential accumulation of LAT is not due to preferential LAT promoter activity in different types of neurons (11). Through the use of viral chimerae, we mapped the function(s) responsible for differential establishment of latency of HSV in A5+ and KH10+ neurons to the LAT region [2], [3]. In the present studies, we have presented evidence that mechanisms responsible for LAT-mediated differential establishment of latency *in vivo* are likely *cis*-acting functions, since co-infection of mice with HSV-1 and HSV-2 did not alter preferential establishment of latent infection. We have further demonstrated that the mechanisms that regulate differential permissiveness for productive infection *in vitro* are also likely to be *cis*-acting in nature, since HSV-1 and HSV-2 do not appear to produce factors that act independently in *trans* to regulate preferential permissiveness for productive infection in A5+ and KH10+ adult trigeminal neurons *in vitro*. While there are neuronal populations that support productive infection of both HSV-1 and HSV-2 (25.2% of neurons in culture were productively infected with both HSV-1 and HSV-2), there are also populations in which each virus cannot efficiently express lytic cycle genes. A5+ neurons do not support productive infection of HSV-1 and infection of these neurons results in latent infection representing half of the latent reservoir *in vivo* [1]–[3]. Similarly, KH10+ neurons do not support productive infection with HSV-2, which leads to latent infection in this population [2], [3], also representing half of the latent HSV-2 reservoir *in vivo*. Thus, approximately half of the latent HSV reservoir appears to be maintained in neurons incapable of supporting productive infection. Populations of neurons other than A5+ or KH10+ neurons make up about 75% of all of the neurons in the TG, and as a group these populations appear to be capable of maintaining latent HSV and also supporting productive infection of the virus, and thus may serve as the reservoir for reactivation-competent HSV.

The role of the HSV LAT region in the establishment and maintenance of latent infection has been extensively investigated, largely through the use of deletion viruses. The mechanisms by which this region accomplishes these functions are less well understood, but include effects on IE gene expression, heterochromatin formation, and apoptosis [5], [16]–[19]. A number of RNAs are expressed from this region, including microRNAs, the stable LAT intron, and RNAs antisense to LAT [9], [11], [20], [21]. Our data suggest that the RNAs that are transcribed from the 2.8 kb of the LAT region investigated in our studies do not play a direct *trans*-acting role in the establishment of latent infection of the heterologous HSV serotype in distinct neuronal populations. However, it is possible that *trans*-acting factors expressed from this region are not functional in the heterologous serotype or that specific *trans*-acting factors may not be expressed in all neuronal populations. It is also possible that previously identified transcripts expressed from this LAT region are important for functions other than neuronal specificity.

A theory on how the LAT region of the HSV genome might act in a *cis*–regulated manner to transcriptionally regulate key lytic gene promoters has been previously proposed by one of the authors (3). To summarize, Bloom and colleagues proposed that the B1 and B2 CTCF insulators in the 5′ LAT region might interact, forming a loop domain containing the LAT enhancer. Formation of this DNA loop would enable the LAT enhancer to interact with local promoters that regulate key immediate early gene expression, such as those involved in the transcription of ICP4, ICP0, and ICP27. Furthermore, the transcriptional outcome of any given LAT enhancer/IE promoter interaction (positive or negative) would depend on a given cell’s transcriptional milieu, which is clearly different amongst different types of TG sensory neurons.

The studies described herein provide an important piece of the puzzle related to HSV pathogenesis; the HSV-1 and HSV-2 2.8 kb LAT regions investigated in these studies do not produce *trans*-acting factors that regulate preferential viral behavior in different types of neurons. However, co-infection with HSV-1 and HSV-2 in the murine ocular infection model presents several challenges that limit the interpretations of the results from these studies. Ocular infection of mice with HSV-1 strain 17syn+ or any HSV-2 strain requires post-infection treatment with acyclovir to prevent undue mortality rates, although we determined in previous studies that acyclovir treatment starting at 40 hpi does not significantly alter the patterns of preferential establishment of latent infection. In the current studies, co-infection with any strain of HSV-1 and any strain of HSV-2 increased the probability of mortality despite treatment with acyclovir, thus limiting the range of viral inocula we were able to investigate, particularly after co-infection with HSV-1 17syn+ and HSV-2 333. The high mortality rate of mice co-infected with HSV-1 and HSV-2 suggests that these viruses likely interact in some manner, but an interaction was not evident in ganglionic A5+ and KH10+ neurons, in which half of the latent HSV-1 and HSV-2 reservoirs are detected, respectively [1]–[3]. HSV-1 and HSV-2 also demonstrate different kinetics during acute infection, both *in vivo* and *in vitro*, which may have permitted one virus to out-compete the other in specific types of neurons. Although we tested staggered infection *in vitro* with no differences in outcome (unpublished observations), our *in vitro* infections were carried out in the absence of an adaptive immune response and in the absence of the normal three-dimensional structure of the trigeminal ganglion.

Taken together, our results to date show that 1) certain populations of trigeminal ganglion neurons are more likely to support productive infection in an HSV-serotype-specific manner, which results in the establishment of HSV latent infection in a serotype-specific manner [1]–[4]; 2) there is a viral function that regulates this set of phenotypes and it appears to map to the LAT region of the viral genome [2], [3]; and 3) this function is not mediated in *trans* by factors produced by the 2.8 kb region of the HSV-1 and HSV-2 LAT regions investigated in these studies. Although exchanging this 2.8 kb region of the LAT between HSV-1 and HSV-2 also exchanges viral preferences for productive or latent infection in specific types of trigeminal neurons, the mechanism by which this occurs is not *trans*-acting.
